# The Efficacy of 308‐nm Excimer Laser With TopicalBimatoprost 0.03% for Facial Vitiligo

**DOI:** 10.1111/jocd.70020

**Published:** 2025-02-19

**Authors:** Maryam Ghiasi, Ahdie Isazade, Tahereh Marhamati, Vahideh Lajevardi, Safoura Shakoei

**Affiliations:** ^1^ Department of Dermatology, Razi Hospital Tehran University of Medical Sciences (TUMS) Tehran Iran; ^2^ Shahid Beheshti University of Medical Sciences Tehran Iran; ^3^ Department of Dermatology Imam Khomeini Hospital Complex, Tehran University of Medical Sciences (TUMS) Tehran Iran

**Keywords:** Bimatoprost, excimer laser, vitiligo

## Abstract

**Background:**

Vitiligo is a commonly acquired autoimmune pigmentary disorder. Some patients resist conventional treatments, leading to the search for combination therapies.

**Aims:**

This study aimed to compare the efficacy of 308‐nm excimer laser monotherapy versus combined treatment with topical bimatoprost 0.03% in patients with facial vitiligo.

**Patients and Methods:**

A single‐blind randomized clinical trial was conducted at Razi Hospital, Tehran, on 38 patients with facial vitiligo who had at least one facial vitiligo patch and had not undergone treatment in the past 3 months. The patients were randomly allocated to either the intervention group (*n* = 18) or the control group (*n* = 20). Both groups received 308‐nm EL therapy twice weekly for 15 weeks, while the intervention group additionally applied 0.03% bimatoprost solution daily. The patients were visited at the end of every 5 weeks and after the 15th week. Efficacy was evaluated using the scale for improvement assessment (SAI), visual analog scale satisfaction (VASS), and visual analog scale improvement (VASI). Data analysis was performed using the Mann–Whitney and *t*‐tests, with a significance level set at *p* < 0.05.

**Results:**

Of the total patients, 27 (71.05%) were female and 11 (28.95%) were male. The male‐to‐female ratio was 50% in the intervention group and 33.33% in the control group. The mean VASI score, as the primary outcome, showed a significant increase over time in the intervention group (*p* ≤ 0.001), increasing from 4.53 to 7.20 (an increase of 2.67 units). Additionally, the intervention had a significant effect on the VASI outcome compared to the control (*p* ≤ 0.001; mean difference: 2.55 [1.63 to 3.47]). As the secondary outcomes, the mean VASS and SAI scores significantly decreased over time in the intervention group (*p* ≤ 0.05). The intervention also significantly affected the VASS and SAI outcomes compared to the control (*p* ≤ 0.001). The side effects of the intervention group were hypertrichosis in three patients and erythema burning in one patient.

**Conclusions:**

Adding bimatoprost 0.03% to the treatment regimen may improve the outcomes of patients with facial vitiligo who are resistant to conventional treatments.

## 1 Introduction

Vitiligo is an autoimmune disease characterized by depigmented patches due to the loss of melanin [1, 2]. Its worldwide prevalence ranges from 0.05% to 2% [3, 4, 5]. Vitiligo is recognized as a systemic condition linked to various organ‐specific and systemic disorders, including ocular and otologic diseases, autoimmune conditions, metabolic syndrome, and psychological issues [6]. About 60% of vitiligo patients have been reported to suffer from mental disorders [7]. Studies have revealed that it can have a negative impact on the patient's quality of life [8], causing anxiety disorders, depression, and low self‐confidence [9, 10]. Factors associated with a significantly higher burden included female sex, visible or genital lesions, age < 30 years (mostly adolescents), and greater body surface area involvement, among others [11]. In addition, the damage to intimate relationships and sexual performance in affected females is more considerable than that in healthy females [12].

There are several different surgical and nonsurgical treatments for vitiligo [13], ranging from phototherapy, topical and systemic corticosteroids [14], vitamin D analogs [15], calcineurin inhibitors, basic fibroblast growth factor (bFGF) derived peptide [16], and camouflage techniques or depigmenting agents to combined surgical procedures [17, 18].

The 308 nm excimer laser (EL) is an FDA‐approved phototherapy device widely used in clinical practice for treating vitiligo [19]. It offers high precision and the ability to deliver a specific wavelength (308 nm) of radiation to target tissues efficiently over a short period. The EL has movable beam transmission capabilities that allow for selective light delivery to the specific lesion while maintaining healthy skin, thus reducing the risk of erythema in the surrounding depigmented area from overexposure, a common side effect in other phototherapy procedures [20, 21]. While some treatments can be successful for particular patients, some patients resist conventional therapies [22], possibly due to this disease's multifactorial and multigenic nature, motivating the search for new combination therapies [23]. Topical steroids are the first line of treatment for vitiligo, but they may be associated with more side effects in the facial area due to the cosmetic importance of appearance [24]. Bimatoprost solution 0.03%, a prostaglandin‐ethanolamide F2α (PGF2α) analogue, has been studied for its potential in treating vitiligo due to its ability to induce melanogenesis. Pharmacological and clinical research has shown it to be a safe and effective alternative therapy for this condition [25].

Therefore, this study aims to assess whether combining 308 nm EL therapy with bimatoprost 0.03% solution enhances treatment outcomes in patients with facial vitiligo compared to using EL therapy alone.

## 2 Materials and Methods

This study is a single‐blind randomized clinical trial conducted on the selected patients from Razi Hospital of Tehran between 2021 and 2022. We recruited vitiligo patients over 20 years old who had at least one facial vitiligo patch and had not undergone treatment in the past 3 months. Exclusion criteria included pregnant or nursing women, patients with a history of photosensitivity or allergy to bimatoprost, and those who experienced complications with bimatoprost or laser therapy. G‐Power software was used to determine the sample size. The Determine option was used to determine the effect size. In this software, taking into account the ANOVA test and assuming an effect size of 0.5based on the study and the significance level of 0.05, a power of 80% was obtained for two groups; by measuring three times, the required sample volume of 38 patients was obtained in both groups. Considering a 10% loss to follow‐up, the total sample size was 42 patients. A total of 38 patients who were given sufficient information about the experiments and signed informed consent were included in the study. Then, they were randomly allocated to the intervention and control groups, with 18 and 20 patients, respectively. Using blocks of 3, 6, and 9, the random block method has been used to randomize. Information about the grouping and executive procedure is not given to the patients to blind them. The researcher and analyst will also receive information from groups A and B.

At the beginning of the study, the demographic characteristics of the patients, including age, gender, occupation, etc., were obtained. Both groups of patients were treated with 308 nm EL twice a week for 15 weeks. The dose of 308 nm EL started at 100 (mj/cm^2^) and continued by adding 10% to the first dose every session. In addition, the intervention group applied 0.03% bimatoprost solution (Excilia, Inoclon, Tehran, Iran) once daily for 15 weeks. To monitor adherence to the bimatoprost treatment, patients were provided with a treatment diary to document daily application, and the remaining solution volume was checked at each follow‐up visit. Noncompliance was recorded when more than three missed applications per week were reported. The efficacy of the treatments was evaluated using the visual analog scale for improvement (VASI), which assesses the percentage of repigmentation from the patient's perspective; the visual analog scale for satisfaction (VASS), which measures overall patient satisfaction with the treatment; and the scale for improvement assessment (SAI), a physician‐assessed scale that objectively evaluates the degree of repigmentation based on clinical examination, considering factors such as the size, distribution, and color match of the repigmented areas. We standardized the EL treatment protocol across both groups to minimize procedural differences and reduce potential bias. The study staff and physicians conducting the assessments were blinded to the treatment group assignments, thereby reducing bias in evaluating outcomes. The intervention group received both bimatoprost solution and excimer laser, while the control group received only excimer laser, so single‐blinding was challenging. Photographs were taken at the beginning of the study, with follow‐up evaluations every 5 weeks and at the end of the 15‐week treatment period. Changes in patient condition were assessed through photographic records by two individual physicians, with any discrepancies resolved by a third physician. Patients who encountered complications due to bimatoprost or laser therapy or required a change in treatment due to disease exacerbation were excluded.

The Shapiro–Wilk test was employed to assess the normality of the primary outcome. Continuous variables were expressed either as mean ± standard deviation. Categorical variables were presented as frequencies (%). A multivariate analysis of variance and covariance was employed to compare and determine significant differences between the means of the primary outcome in this study across two groups. The interaction of time and group was assessed through multivariate analysis of variance and covariance (adjusted for baseline variable and age). The statistical software Stata (Version 18, Stata Corp, College Station, Texas, USA) was used for data analysis. Statistical significance was defined as a *p*‐value less than 0.05.

## 3 Results

The presented data in Table 1 show that out of the total patients, 27 (71.05%) were female and 11 (28.95%) were male. The male‐to‐female ratio was 50% in the intervention group and 33.33% in the control group. The mean age of subjects in the intervention group was 25.16 ± 9.80, and in the control group was 32.45 ± 11.53, indicating no significant difference between the two groups (*p* = 0.06). Although the disease duration in the control group (6.95 ± 5.24 years) was longer than that in the intervention group, the difference was not significant (*p* > 0.05).

**TABLE 1:** Baseline characteristics of study participants. Data are frequencies (%) unless specified differently.

	Comparison group	Intervention group	*p*
*N*	18 (46.0)	20 (54.0)	
Age (year), mean (SD)	32.45 ± 11.53	25.16 ± 9.80	0.061
Sex			
Male	5 (25%)	5 (33%)	0.572
Female	15 (75%)	12 (66%)	
Duration of disease (year), mean (SD)	6.95 ± 5.24	4.64 ± 2.08	0.201
Fitzpatrick skin type, *N* (%)			
Type 1	0	0	0.723
Type 2	1 (5.00%)	1 (5.88%)	
Type 3	15 (75.00%)	13 (76.47%)	
Type 4	4 (20.00%)	3 (17.64%)	
Type 5	0 (0.00%)	0 (0.00%)	

Abbreviations: *N*, number; SD, standard deviation.

The findings in Table 2 show that the three outcomes, including VASI (*p* ≤ 0.001), VASS (*p* = 0.003), and SAI (*p* = 0.002) increased significantly over time. The average scores for the measures over time showed that VASI, as the primary outcome, after the 10th treatment session was higher in the intervention group than in the control group (*p* = 0.002), whereas VASS in the 20th (*p* = 0.002) and 30th (*p* ≤ 0.001) sessions was greater in the intervention group compared to the control group (Table 3).

**TABLE 2:** Comparison of measurement scores after sessions 10, 20, and 30 in each group.

Outcomes	Control group (mean ± SD)	Intervention group (mean ± SD)
VASI		
Session 10	2.78 ± 2.12	4.53 ± 1.12
Session 20	4.12 ± 2.57	6.00 ± 1.22
Session 30	4.61 ± 3.09	7.20 ± 1.31
*p* *	0.118	< 0.001
VASS		
Session 10	3.26 ± 3.21	5.00 ± 1.64
Session 20	4.56 ± 3.55	6.38 ± 1.55
Session 30	5.00 ± 3.67	7.30 ± 1.49
*p*	0.331	0.003
SAI		
Session 10	2.47 ± 1.26	3.60 ± 0.98
Session 20	3.26 ± 1.75	4.23 ± 0.72
Session 30	3.58 ± 2.10	5.00 ± 0.94
*p*	0.181	0.002

* All *p* in the table were calculated by MANOVA.

Abbreviations: SAI, scale for improvement assessment; SD, standard deviation; VASI, visual analog scale improvement; VASS, visual analog scale satisfaction.

**TABLE 3:** The effect of intervention and its interaction with time on the outcomes (adjusted for disease duration and age).

Outcome	Pairwise comparison	Mean difference (95% CI)	*p* (pairwise)	*p* * (MANOVA)
VASI				
Group	Intervention vs. control	2.55 (1.63 to 3.47)	≤ 0.001	≤ 0.001
Interaction of time and group	Intervention vs. control at 10th	2.19 (0.81 to 3.58)	0.002	0.607
	Intervention vs. control at 20th	2.41 (0.91 to 3.92)	0.002	
	Intervention vs. control at 30th	3.24 (1.53 to 4.95)	≤ 0.001	
VASS				
Group	Intervention vs. control	2.50 (1.23 to 3.77)	≤ 0.001	≤ 0.001
Interaction of time and group	Intervention vs. control at 10th	2.22 (0.81 to 3.58)	0.023	0.850
	Intervention vs. control at 20th	2.39 (0.32 to 4.47)	0.024	
	Intervention vs. control at 30th	3.04 (0.68 to 5.41)	0.012	
SAI				
Group	Intervention vs. control	1.40 (0.75 to 2.04)	≤ 0.001	≤ 0.001
Interaction of time and group	Intervention vs. control at 10th	1.32 (0.36 to 2.28)	0.008	0.765
	Intervention vs. control at 30th	1.20 (0.14 to 2.26)	0.027	
	Intervention vs. control at 30th	1.75 (0.56 to 2.93)	0.004	

* Adjusted for duration of disease and age and calculated by MANOVA.

Abbreviations: CI, confidence interval for the difference in means; SAI, scale for improvement assessment. SD, standard deviation, VASI, visual analog scale improvement; VASS, visual analog scale satisfaction.

**FIGURE 1:**
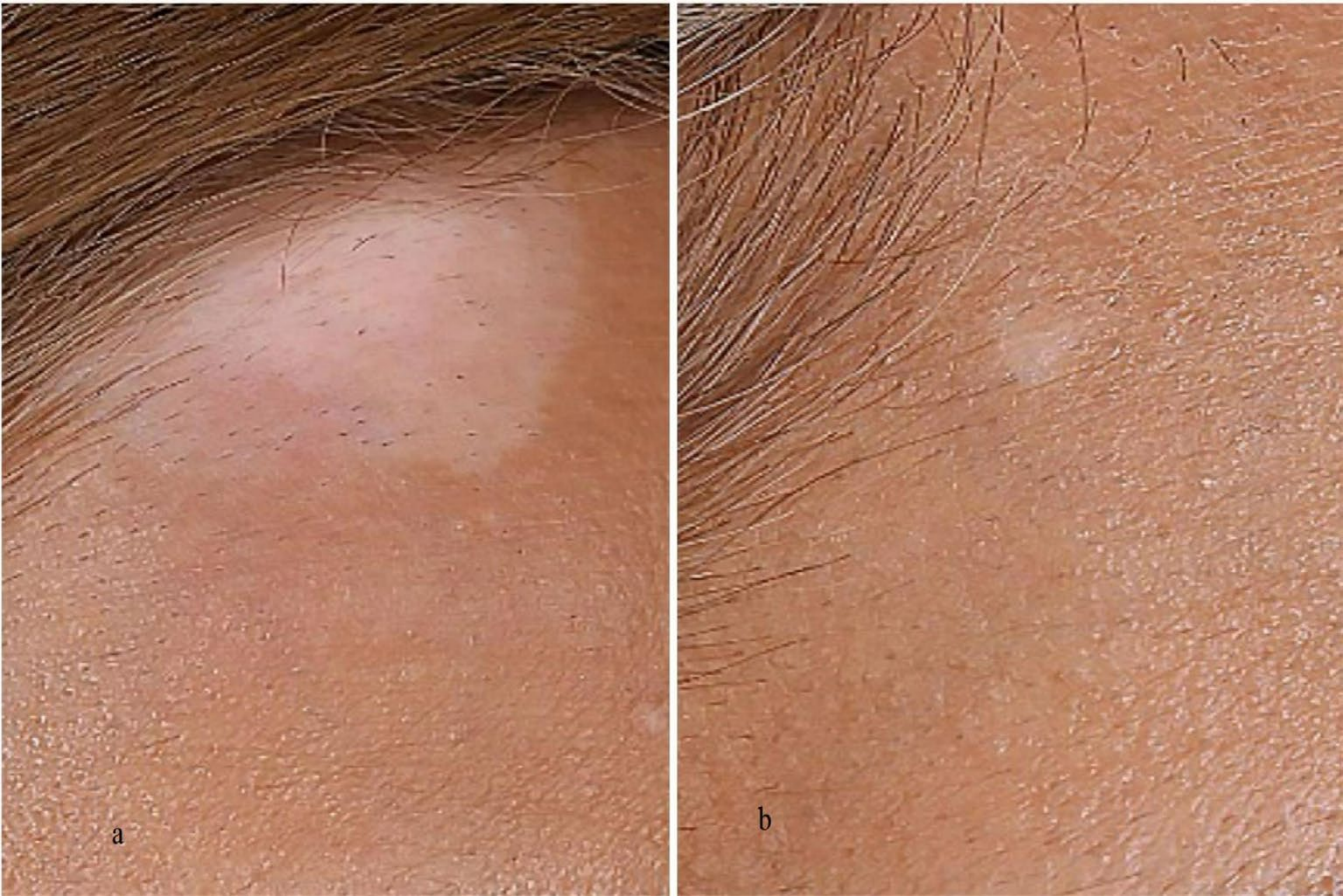
Representative patient before and after 15 weeks of treatment with the combination of 308‐nm excimer laser and topical bimatoprost 0.03%. The figure shows the noticeable repigmentation of facial vitiligo patches as a result of the combination therapy. The left side of the image depicts the patient before treatment, whereas the right side shows the improved pigmentation after the 15‐week treatment period.

Based on the MANOVA analyses, the intervention had a significant effect on the VASI (*p* ≤ 0.001; mean difference: 2.55 [1.63 to 3.47]), the VASS outcome (*p* ≤ 0.001; mean difference: 2.50 [1.23 to 3.77]) and the SAI outcome (*p* ≤ 0.001; mean difference: 1.40 [0.75 to 2.04]) when compared to the control group (Figure 1). Additionally, the interaction effect of time and group was not significant for all outcomes (*p* > 0.05). In the control group, side effects appeared in two patients: one experienced erythema burning, and another developed erythema. The side effects of the intervention group were hypertrichosis in three patients and erythema burning in one patient.

## 4 Discussion

The results of this study suggest that the combination therapy of 308‐nm excimer laser and topical bimatoprost 0.03% yields superior outcomes compared to excimer laser monotherapy for treating facial vitiligo. This indicates a more effective improvement and patient satisfaction with the combination therapy. The lack of significant differences in side effect occurrence between the two groups implies that adding bimatoprost does not increase the risk of adverse effects compared to excimer laser alone. The disease duration was similar between the groups, suggesting that the observed differences in efficacy are not confounded by disease duration.

The best options for treating vitiligo patients depend on factors such as age, patient condition and expectations, distribution and size of depigmentation, type of vitiligo (stable or unstable), availability, and the cost of interventions [26]. Several contributing factors, including male gender, age of 15 years or less, and disease duration of 2 years or less, are associated with increased pigmentation [27]. Facial lesions and younger patients showed better responses to treatments [28, 29, 30].

However, the therapeutic effect of EL as a well‐established procedure on vitiligo was reported for the first time in 2001 [31]. Since then, most studies have reported the regimentation effect of this form of phototherapy [31, 32]. Many studies have suggested that combination therapy has superior efficacy relative to monotherapy. These have focused on EL combination therapies involving tacrolimus o.1% [33, 34], antioxidants [35], glucocorticoids [36], and platelet‐rich plasma (PRP) [37]. Li and colleagues reported that patients undergoing a combination treatment of EL plus halometasone experienced greater regimentation [38]. Other studies have confirmed that EL, combined with PRP, appears to be more effective than EL alone in treating vitiligo [39, 40]. In one study by Ju et al., the combination therapy of EL and a topical immunosuppressant in a large cohort of patients has been suggested [41]. Noncultured epidermal cell suspension (NCECS) combined with excimer lamps increased treatment efficacy to 100% compared to only NCECS [42].

Bimatoprost is a synthetic PGF2α analogue. Prostaglandins are lipid compounds produced by cells involved in inflammation, tissue damage, or infectious processes. Previous studies showed that both PGE2 and PGF2α induced hyperpigmentation via melanocytic activation [25]. In addition, research has shown that PGF2α analogs, such as bimatoprost, significantly increase tyrosinase enzyme activity, mainly when used with ultraviolet irradiation [43, 44]. Furthermore, prior investigations indicate that combining bimatoprost with other treatments, such as 1550 nm fractional laser and narrowband ultraviolet B (NB‐UVB) phototherapy, yields more effective results than monotherapy [25, 45]. However, Abdhelrazik et al. (2020) concluded that fractional CO2 laser followed by topical calcipotriol (0.05 mg/g) along with betamethasone ointment (0.5 mg) had superior efficacy compared with fractional CO2 laser combined with topical bimatoprost (0.03%) in the treatment of vitiligo [46]. Nonetheless, despite the approval of bimatoprost's therapeutic effect in treating vitiligo, some studies have suggested further large‐scale work on this topic [47, 48].

Hypertrichosis can be seen in 1%–10% of the patients treated with bimatoprost. The changes persist for varying periods but are reversible [49].

Our study corroborates previous research suggesting that while side effects are generally mild and comparable between treatment modalities, the combination therapy of excimer laser with bimatoprost offers significant advantages in terms of efficacy without additional safety concerns. This finding supports the use of combination therapy as a more effective approach for treating facial vitiligo [25, 39, 40, 48].

This study has several limitations that should be acknowledged. Firstly, the relatively small sample size of 38 patients may limit the generalizability of our findings. Although the sample size was calculated to ensure adequate statistical power, a larger cohort could provide more robust evidence and improve the applicability of the results to a broader patient population. Secondly, the study was conducted at a single institution, which may introduce site‐specific biases and limit the external validity of the results. Thirdly, the study duration of 15 weeks may not capture the long‐term efficacy and safety of the combination therapy, necessitating future research with more extensive, multicenter trials and extended follow‐up periods to better understand the long‐term outcomes of excimer laser combined with bimatoprost for treating facial vitiligo. Finally, achieving complete blinding of patients was challenging. The intervention group received both bimatoprost solution and excimer laser, while the control group received only excimer laser. Despite efforts to standardize the excimer laser treatment protocol and blind the study staff and physicians conducting assessments, the potential for unintentional bias due to patients' awareness of their treatment assignment remains a concern, which may have influenced patient condition. We recommend using a placebo of bimatoprost in the comparison group for future studies, as this approach helps differentiate the actual effects of the treatment from the placebo effect, providing a clearer understanding of patient satisfaction with the treatment.

## 5 Conclusion

Combining 308‐nm EL with topical bimatoprost 0.03% significantly enhances treatment outcomes for facial vitiligo, as evidenced by higher VASI, VASS, and SAI scores compared to EL alone. This indicates superior repigmentation and greater patient‐reported satisfaction with the combination therapy. Clinicians should consider this approach to achieve more effective and satisfactory results. Future research involving more extensive, multi‐center trials and extended follow‐up is needed to confirm these findings and evaluate the long‐term benefits and safety of this combined therapy.

## Author Contributions

Study concept, design, and technical supervision: Maryam Ghiasi, Safoura Shakoei, and Vahideh Lajevardi. Acquisition of data and drafting of the manuscript: Ahdie Isazade. Writing – original DraftPreparation: Tahereh Marhamati. Critical revision of the manuscript: Safoura Shakoei. Supervision: Maryam Ghiasi. All authors have reviewed and approved the article for submission.

## Disclosure

The authors have nothing to report.

## Ethics Statement

The study protocol was approved by the Research Ethics Committee of Tehran Medical University with the reference number of (IR.TUMS.MEDICINE.REC.1399.610), and was performed in accordance with the ethical standards as laid down in the 1964 Declaration of Helsinki and its later amendments or comparable ethical standards.

## Conflicts of Interest

Topical bimatoprost 0.03% solution (Excilia, Inoclon, Tehran, Iran) was supplied by Inoclon Company.

## Data Availability

The data that support the findings of this study are available on request from the corresponding author. The data are not publicly available due to privacy or ethical restrictions.

## References

[1] N. Rezaei , N. G. Gavalas , A. P. Weetman , and E. H. Kemp , “Autoimmunity as an Aetiological Factor in Vitiligo,” Journal of the European Academy of Dermatology and Venereology 21, no. 7 (2007): 865–876, 10.1111/j.1468-3083.2007.02228.x. 17658994

[2] M. Imran , V. R , and Y. Mohammed , “Vitiligo: A Review of Aetiology, Pathogenesis, Treatment, and Psychosocial Impact,” Cosmetics 10, no. 3 (2023): 84.

[3] C. Krüger and K. U. Schallreuter , “A Review of the Worldwide Prevalence of Vitiligo in Children/Adolescents and Adults,” International Journal of Dermatology 51, no. 10 (2012): 1206–1212, 10.1111/j.1365-4632.2011.05377.x. 22458952

[4] Y. Zhang , Y. Cai , M. Shi , et al., “The Prevalence of Vitiligo: A Meta‐Analysis,” PLoS One 11, no. 9 (2016): e0163806, 10.1371/journal.pone.0163806. 27673680 PMC5038943

[5] K. Gandhi , K. Ezzedine , K. P. Anastassopoulos , et al., “Prevalence of Vitiligo Among Adults in the United States,” JAMA Dermatology 158, no. 1 (2022): 43–50, 10.1001/jamadermatol.2021.4724. 34787670 PMC8600454

[6] Z. Hu and T. Wang , “Beyond Skin White Spots: Vitiligo and Associated Comorbidities,” Frontiers in Medicine 10 (2023): 1072837, 10.3389/fmed.2023.1072837. 36910477 PMC9995999

[7] S. Sarkar , T. Sarkar , A. Sarkar , and S. Das , “Vitiligo and Psychiatric Morbidity: A Profile From a Vitiligo Clinic of a Rural‐Based Tertiary Care Center of Eastern India,” Indian Journal of Dermatology 63, no. 4 (2018): 281–284, 10.4103/ijd.IJD_142_18. 30078869 PMC6052755

[8] S. P. Sajan C , R. Mahajan , V. Chandrakar , D. Baxi , and H. Patel , “Assessment of Quality of Life in Vitiligo Patients in Terms of Clinical Severity and Psychological Burden in a Tertiary Care Hospital: An Observational Study.An Observational Study,” Journal of the Scientific Society 50, no. 1 (2023): 45.

[9] A. K. Rzepecki , B. N. McLellan , and N. Elbuluk , “Beyond Traditional Treatment: The Importance of Psychosocial Therapy in Vitiligo,” Journal of Drugs in Dermatology: JDD 17, no. 6 (2018): 688–691. 29879259

[10] P. E. Grimes and R. Nashawati , “Depigmentation Therapies for Vitiligo,” Dermatologic Clinics 35, no. 2 (2017): 219–227, 10.1016/j.det.2016.11.010. 28317530

[11] K. Ezzedine , V. Eleftheriadou , H. Jones , et al., “Psychosocial Effects of Vitiligo: A Systematic Literature Review,” American Journal of Clinical Dermatology 22, no. 6 (2021): 757–774, 10.1007/s40257-021-00631-6. 34554406 PMC8566637

[12] K. Bonotis , K. Pantelis , S. Karaoulanis , et al., “Investigation of Factors Associated With Health‐Related Quality of Life and Psychological Distress in Vitiligo,” Journal of the German Society of Dermatology: JDDG 14, no. 1 (2016): 45–49, 10.1111/ddg.12729. 26713637

[13] Y. Feng and Y. Lu , “Advances in Vitiligo: Update on Therapeutic Targets,” Frontiers in Immunology 13 (2022): 986918, 10.3389/fimmu.2022.986918. 36119071 PMC9471423

[14] D. E. Kubelis‐López , N. A. Zapata‐Salazar , S. L. Said‐Fernández , et al., “Updates and New Medical Treatments for Vitiligo (Review),” Experimental and Therapeutic Medicine 22, no. 2 (2021): 797, 10.3892/etm.2021.10229. 34093753 PMC8170669

[15] B. S. Daniel and R. Wittal , “Vitiligo Treatment Update,” Australasian Journal of Dermatology 56, no. 2 (2015): 85–92, 10.1111/ajd.12256. 25495880

[16] K. Agarwal , I. Podder , M. Kassir , et al., “Therapeutic Options in Vitiligo With Special Emphasis on Immunomodulators: A Comprehensive Update With Review of Literature,” Dermatologic Therapy 33, no. 2 (2020): e13215, 10.1111/dth.13215. 31891450

[17] A. Taieb , A. Alomar , M. Böhm , et al., “Guidelines for the Management of Vitiligo: The European Dermatology Forum Consensus,” British Journal of Dermatology 168, no. 1 (2013): 5–19, 10.1111/j.1365-2133.2012.11197.x. 22860621

[18] A. Frączek , M. Kasprowicz‐Furmańczyk , W. Placek , and A. Owczarczyk‐Saczonek , “Surgical Treatment of Vitiligo,” International Journal of Environmental Research and Public Health 19, no. 8 (2022): 4812, 10.3390/ijerph19084812. 35457678 PMC9031570

[19] C. Guo , X. Gu , J. Zhang , et al., “Efficacy of Fire Needle Combined With 308 Nm Excimer Laser Therapy for Vitiligo: A Systematic Review and Meta‐Analysis of Randomized Controlled Trials,” Journal of Cosmetic Dermatology 23, no. 8 (2024): 2592–2602, 10.1111/jocd.16308. 38591186

[20] N. Silpa‐Archa , H. W. Lim , and C. Wongpraparut , “Excimer Laser in Vitiligo: Where There Is Light, There Is Hope,” British Journal of Dermatology 181, no. 1 (2019): 21–22, 10.1111/bjd.18101. 31259412

[21] A. Paro Vidolin , C. Aurizi , and G. Leone , “Phototherapy for Vitiligo, What's New?,” Giornale Italiano di Dermatologia e Venereologia: Organo Ufficiale, Societa Italiana di Dermatologia e Sifilografia 152, no. 5 (2017): 474–488, 10.23736/s0392-0488.17.05721-2. 28906087

[22] K. Toriyama , H. Kato , H. Sato , T. Tanaka , M. Inoie , and A. Morita , “Cultured Epidermal Autografts for Treatment of Stable Vitiligo: Quantitative Analysis of Color Matching With Surrounding Normally Pigmented Skin,” Journal of Dermatology 48, no. 9 (2021): 1405–1408, 10.1111/1346-8138.16028. 34169570 PMC8453891

[23] M. Picardo and E. Bastonini , “A New View of Vitiligo: Looking at Normal‐Appearing Skin,” Journal of Investigative Dermatology 135, no. 7 (2015): 1713–1714, 10.1038/jid.2015.92. 26066890

[24] D. J. Gawkrodger , A. D. Ormerod , L. Shaw , et al., “Guideline for the Diagnosis and Management of Vitiligo,” British Journal of Dermatology 159, no. 5 (2008): 1051–1076, 10.1111/j.1365-2133.2008.08881.x. 19036036

[25] N. Silpa‐Archa , S. Likittanasombat , C. Apinuntham , et al., “The Efficacy of Bimatoprost Ophthalmic Solution Combined With NB‐UVB Phototherapy in Non‐Segmental and Segmental Vitiligo: A Single‐Blind Randomized Controlled Study,” Scientific Reports 13, no. 1 (2023): 6438, 10.1038/s41598-023-32591-8. 37081101 PMC10119098

[26] M. Bertolani , E. Rodighiero , M. B. de Felici Del Giudice , T. Lotti , C. Feliciani , and F. Satolli , “Vitiligo: What's Old, What's New,” Dermatology Reports 13, no. 2 (2021): 9142, 10.4081/dr.2021.9142. 34659674 PMC8451070

[27] S. M. Kim , H. S. Lee , and S. K. Hann , “The Efficacy of Low‐Dose Oral Corticosteroids in the Treatment of Vitiligo Patients,” International Journal of Dermatology 38, no. 7 (1999): 546–550, 10.1046/j.1365-4362.1999.00623.x. 10440289

[28] J. M. Bae , B. Y. Hong , J. H. Lee , J. H. Lee , and G. M. Kim , “The Efficacy of 308‐Nm Excimer Laser/Light (EL) and Topical Agent Combination Therapy Versus EL Monotherapy for Vitiligo: A Systematic Review and Meta‐Analysis of Randomized Controlled Trials (RCTs),” Journal of the American Academy of Dermatology 74, no. 5 (2016): 907–915, 10.1016/j.jaad.2015.11.044. 26785803

[29] Y. Fa , Y. Lin , X. J. Chi , et al., “Treatment of Vitiligo With 308‐Nm Excimer Laser: Our Experience From a 2‐Year Follow‐Up of 979 Chinese Patients,” Journal of the European Academy of Dermatology and Venereology: JEADV 31, no. 2 (2017): 337–340, 10.1111/jdv.13917. 27538097

[30] N. Ostovari , T. Passeron , W. Zakaria , et al., “Treatment of Vitiligo by 308‐Nm Excimer Laser: An Evaluation of Variables Affecting Treatment Response,” Lasers in Surgery and Medicine 35, no. 2 (2004): 152–156, 10.1002/lsm.20057. 15334620

[31] E. Baltás , Z. Csoma , F. Ignácz , A. Dobozy , and L. Kemény , “Treatment of Vitiligo With the 308‐Nm Xenon Chloride Excimer Laser,” Archives of Dermatology 138, no. 12 (2002): 1619–1620. 12472364

[32] S. Esmat , R. A. Hegazy , S. Shalaby , S. C. Hu , and C. E. Lan , “Phototherapy and Combination Therapies for Vitiligo,” Dermatologic Clinics 35, no. 2 (2017): 171–192, 10.1016/j.det.2016.11.008. 28317527

[33] D. F. Suo , S. W. Zeng , and L. H. Meng , “308 Nm Excimer Laser and Tacrolimus Ointment in the Treatment of Facial Vitiligo: A Systematic Review and Meta‐Analysis,” Lasers in Medical Science 39, no. 1 (2024): 90, 10.1007/s10103-024-04033-y. 38456924

[34] F. Bapur Erduran and E. Adışen , “Comparison of the Efficacy of 308‐Nm Excimer Lamp Monotherapy With Topical Tacrolimus or Clobetasol 17‐Propionate Combination Therapies in Localized Vitiligo,” Photodermatology, Photoimmunology & Photomedicine 32, no. 5–6 (2016): 247–253, 10.1111/phpp.12266. 27552312

[35] M. Soliman , N. A. Samy , M. Abo Eittah , and M. Hegazy , “Comparative Study Between Excimer Light and Topical Antioxidant Versus Excimer Light Alone for Treatment of Vitiligo,” Journal of Cosmetic and Laser Therapy 18, no. 1 (2016): 7–11, 10.3109/14764172.2015.1052510. 26052813

[36] P. Juntongjin and N. Toncharoenphong , “Effectiveness of a Combined 308‐Nm Excimer Lamp and Topical Mid‐Potent Steroid Treatment for Facial Vitiligo: A Preliminary, Randomized Double‐Blinded Controlled Study,” Lasers in Medical Science 35, no. 9 (2020): 2023–2029, 10.1007/s10103-020-03048-5. 32458080

[37] J. Chen , N. Yu , H. Li , Y. Tang , and H. Zhu , “Meta‐Analysis of the Efficacy of Adding Platelet‐Rich Plasma to 308‐Nm Excimer Laser for Patients With Vitiligo,” Journal of International Medical Research 50, no. 9 (2022): 19646, 10.1177/03000605221119646. PMC9446466 36062405

[38] L. Li , Y. Liang , J. Hong , L. Lan , H. Xiao , and Z. Xie , “The Effectiveness of Topical Therapy Combined With 308‐Nm Excimer Laser on Vitiligo Compared to Excimer Laser Monotherapy in Pediatric Patients,” Pediatric Dermatology 36, no. 1 (2019): e53–e55, 10.1111/pde.13726. 30520111

[39] F. M. Khattab , E. Abdelbary , and M. Fawzi , “Evaluation of Combined Excimer Laser and Platelet‐Rich Plasma for the Treatment of Nonsegmental Vitiligo: A Prospective Comparative Study,” Journal of Cosmetic Dermatology 19, no. 4 (2020): 869–877, 10.1111/jocd.13103. 31541597

[40] Y. Deng , J. Li , and G. Yang , “308‐Nm Excimer Laser Plus Platelet‐Rich Plasma for Treatment of Stable Vitiligo: A Prospective, Randomized Case‐Control Study,” Clinical, Cosmetic and Investigational Dermatology 13 (2020): 461–467, 10.2147/ccid.s260434. 32801821 PMC7398870

[41] H. J. Ju , J. H. Han , M. S. Kim , et al., “The Long‐Term Risk of Lymphoma and Skin Cancer Did Not Increase After Topical Calcineurin Inhibitor Use and Phototherapy in a Cohort of 25,694 Patients With Vitiligo,” Journal of the American Academy of Dermatology 84, no. 6 (2021): 1619–1627, 10.1016/j.jaad.2021.01.067. 33508387

[42] T. Hoang Van , D. Parsad , T. N. Van , et al., “The Efficacy of Non‐Cultured Epidermal Cell Suspension and Excimer Lamps Combination Therapy in Vitiligo: Results of 18 Months Follow‐Up,” Journal of Cosmetic Dermatology (2024): e16714, 10.1111/jocd.16714. 39679850 PMC11845954

[43] M. S. Zaky , R. B. Atallah , N. T. A. El Abasy , and M. L. Elsaie , “Comparative Study Between Efficacy of Excimer Light With Topical Tacrolimus 0.1% Versus Excimer Light With Topical Bimatoprost 0.01% in Treatment of Facial Vitiligo,” Archives of Dermatological Research 316, no. 7 (2024): 350, 10.1007/s00403-024-03054-5. 38850408 PMC11162377

[44] G. Scott , S. Jacobs , S. Leopardi , et al., “Effects of PGF2alpha on Human Melanocytes and Regulation of the FP Receptor by Ultraviolet Radiation,” Experimental Cell Research 304, no. 2 (2005): 407–416, 10.1016/j.yexcr.2004.11.016. 15748887

[45] A. B. Massaki , S. G. Fabi , R. Fitzpatrick , et al., “Repigmentation of Hypopigmented Scars Using an Erbium‐Doped 1,550‐Nm Fractionated Laser and Topical Bimatoprost,” Dermatologic Surgery: Official Publication for American Society for Dermatologic Surgery 38, no. 7 (2012): 995–1001, 10.1111/j.1524-4725.2012.02389.x. 22540767

[46] A. M. Abdelrazik , M. A. El‐Khalawany , and S. M. Ibrahim , “Evaluation of Fractional Carbon Dioxide Laser‐Assisted Drug Delivery of Calcipotriol Plus Betamethasone Versus Bimatoprost in the Treatment of Vitiligo,” Scientific Journal of Al‐Azhar Medical Faculty, Girls 4, no. 2 (2020): 210–216, 10.4103/sjamf.sjamf_23_20.

[47] A. K. Jha , S. Prasad , and R. Sinha , “Bimatoprost Ophthalmic Solution in Facial Vitiligo,” Journal of Cosmetic Dermatology 17, no. 3 (2018): 437–440, 10.1111/jocd.12443. 29034590

[48] A. K. Jha and R. Sinha , “Bimatoprost in Vitiligo,” Clinical and Experimental Dermatology 41, no. 7 (2016): 821–822, 10.1111/ced.12904. 27663165

[49] K. Modschiedler , P. Driesch , and R. Paus , “Hyperpigmentosis and Hypertrichosis of the Eyelids After Use of Bimatoprost Eye Drops,” Journal of the German Society of Dermatology: JDDG 3, no. 4 (2005): 276–277, 10.1111/j.1610-0387.2005.05705.x. 16370476

